# Joint effect of RRP9 and DDX21 on development of colorectal cancer and keloid

**DOI:** 10.18632/aging.205240

**Published:** 2023-11-20

**Authors:** Hao Liu, Xiaoqian Chi, Ning Yang, Mengjie Shan, Yiding Xiao, Mingzi Zhang, Yan Hao, Shiyang Hou, Yabin Liu, Youbin Wang

**Affiliations:** 1 Department of Plastic Surgery, Peking Union Medical College Hospital, Beijing, China; 2 Department of General Surgery, Beijing Rehabilitation Hospital, Capital Medical University, Beijing, China; 3 Department of General Surgery, The First Affiliated Hospital, Hebei Medical University, Shijiazhuang, China; 4 Department of General Surgery, The Fourth Affiliated Hospital, Hebei Medical University, Shijiazhuang, China

**Keywords:** RRP9, DDX21, biomarkers, colorectal cancer, keloid

## Abstract

Background: Colorectal cancer (CRC) is a common malignancy in the gastrointestinal tract. Keloid refers to abnormal scar tissue that forms on the skin or mucous membrane. The relationship between RRP9 and DDX21 and the two diseases is unclear.

Methods: Download the colorectal cancer dataset GSE134834, GSE206800, GSE209892 and keloid dataset GSE44270 from the GEO database. Differentially expressed genes (DEGs) were screened and weighted gene co-expression network analysis (WGCNA) was performed. The construction and analysis of protein–protein interaction (PPI) network, functional enrichment analysis, gene set enrichment analysis (GSEA). Gene expression heat map was drawn. The comparative toxicogenomics database (CTD) analysis was performed to find diseases most related to core genes. TargetScan screened miRNAs that regulated central DEGs. We conducted experimental validation using Western blotting and Polymerase Chain Reaction (PCR).

Results: In the colorectal cancer dataset and the scar tissue dataset, we identified 1380 DEGs and 1000 DEGs, respectively. The enrichment pattern for scar tissue was similar to that of colorectal cancer. We identified two core genes, RRP9 and DDX21. CTD analysis indicated that RRP9 and DDX21 are associated with proliferation, scar tissue, colorectal tumors, scleroderma, and inflammation. We found that the core genes (RRP9 and DDX21) were highly expressed in colorectal cancer and scar tissue samples, while their expression was lower in normal samples. This was further validated through Western blotting (WB) and Polymerase Chain Reaction (PCR).

Conclusions: The higher the expression of RRP9 and DDX21 in colorectal cancer and keloid, the worse the prognosis.

## INTRODUCTION

Colorectal cancer (CRC) is a malignant tumor that occurs in the colon or rectum and has a high mortality rate [1]. The incidence of colorectal cancer in China increased by more than 200 000 cases per year from 1990 to 2012. While most CRC cases are still predominant in western countries, this trend is changing due to rapid development in some countries [2–4]. China, despite its historically low incidence of colorectal cancer, is witnessing an increasing trend in many areas. The disease mainly affects men over middle age, particularly those aged 40 to 70 years old. The male-to-female ratio is approximately 2:1 [5]. Early symptoms of colorectal cancer are often not obvious. As the cancer progresses, symptoms such as changes in bowel habits, bloody stool, and diarrhea may occur. Advanced colorectal cancer is characterized by anemia, weight loss, and other systemic symptoms. Like other types of cancer, mutations in specific genes can contribute to the development of colorectal cancer. Based on the source of gene mutations, colorectal cancer can be categorized as sporadic, sporadic, hereditary, and familial [6–8].

A keloid is an abnormal skin lesion that typically forms during the cell growth and tissue repair processes when the skin is damaged or injured [9]. It results from an excessive response and appears as an elevated area of fibrous tissue on the skin's surface. Keloids can affect individuals of any age, but are more commonly seen in younger people, possibly due to their skin's greater elasticity and healing capabilities [10]. For some individuals, keloids may appear within weeks following the initial injury's healing and gradually recede over time. However, for others, keloids may continue to grow and cause discomfort. Keloids typically manifest as lumps or raised areas of the skin and may be accompanied by varying degrees of itching, pain, or tenderness. Pathologically, they differ in structure and tissue arrangement from normal tissue. While keloids generally do not pose a threat to life, they can lead to discomfort, pain, and itching for affected individuals. In some cases, keloids may impact a person's appearance and self-esteem [11].

RRP9 is a ribosomal RNA processing protein that participates in the maturation process of ribosomal RNA, ensuring the normal formation and function of ribosomes. Additionally, RRP9 is involved in biological processes such as cell cycle regulation and DNA damage repair. In the field of cancer research, abnormal expression of RRP9 has been associated with the occurrence and development of various cancers [12, 13]. In colorectal cancer, studies have found that high expression of RRP9 is associated with enhanced tumor proliferation and invasion capabilities, suggesting a potential role for RRP9 in the development of colorectal cancer [14, 15]. In scar hypertrophy research, certain prognostic biomarkers have been identified. Although the specific mechanisms of the RRP9 gene have not been discussed, RRP9 is known to influence scar hypertrophy formation through various pathways involved in cell cycle regulation.

DDX21 is a member of the DEAD-box protein family and belongs to the RNA helicase category. DDX21 plays a crucial role in RNA-related biological processes, including transcription regulation and maintenance of RNA stability. In the context of cancer, research on DDX21 is relatively new, but studies have indicated that its expression is aberrant in certain tumors, and it is linked to tumor occurrence and progression [15]. In a study by Vina Putra [16] on neuroblastoma, elevated levels of DDX21 were found to be correlated with high levels of N-Myc and CEP55 expression. Moreover, elevated DDX21 expression independently predicted poor prognosis in patients, and knocking out DDX21 led to regression of neuroblastoma tumors in mice and suppressed tumor progression. The formation of keloids is associated with the remodeling of the extracellular matrix and the redistribution of cells [17]. DDX21 may be involved in processes related to transcription regulation and RNA processing, thereby influencing molecular pathways and signal transduction associated with the development of keloids.

Research suggests that RRP9 and DDX21 may play a role in the development of both colorectal cancer and keloids, but their exact molecular mechanisms and regulatory pathways have not been fully elucidated. These proteins may be involved in various pathways that contribute to processes such as cell proliferation, invasion, and metastasis in both diseases, including effects on the cell cycle, gene transcription, and RNA stability. The precise mechanisms by which different genes function in various diseases are also not yet clear.

Therefore, this study aims to utilize bioinformatics techniques to uncover the core genes associated with colorectal cancer, keloids, and normal tissues and conduct an analysis. RRP9 and DDX21's significant roles in colorectal cancer will be validated using public datasets. These two genes' significant roles in colorectal cancer and keloids will also be validated using public datasets, with further validation through basic cellular experiments.

## RESULTS

### Differential gene expression analysis

**Figure 1:**
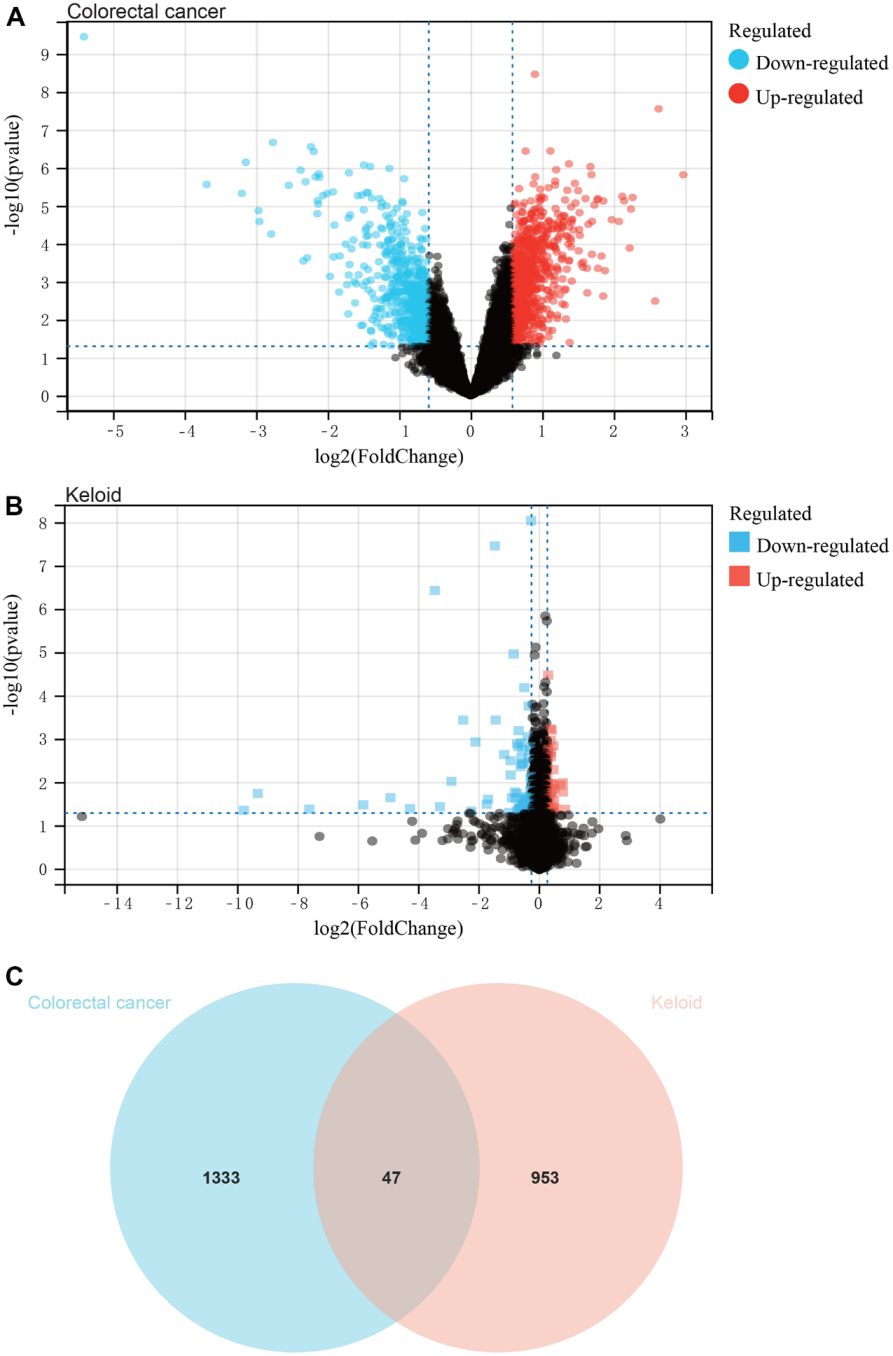
**Analysis of differentially expressed genes.** (**A**) The colorectal cancer dataset, and a total of 1380 DEGs. (**B**) The keloid dataset, and a total of 1000 DEGs. (**C**) The intersection of differential genes of colorectal cancer and keloid was used to obtain the Venn diagram.

In this study, we identified differentially expressed genes (DEGs) in the colorectal cancer datasets GSE134834, GSE206800, and GSE209892 using a predefined cutoff, resulting in a total of 1380 DEGs (Figure 1A). Additionally, we identified 1000 DEGs in the scar tissue dataset GSE44270 (Figure 1B). The Venn diagram (Figure 1C) shows the intersection of DEGs between colorectal cancer and scar tissue datasets, which were used for subsequent analysis.

### Functional enrichment analysis

### DEGs

**Figure 2:**
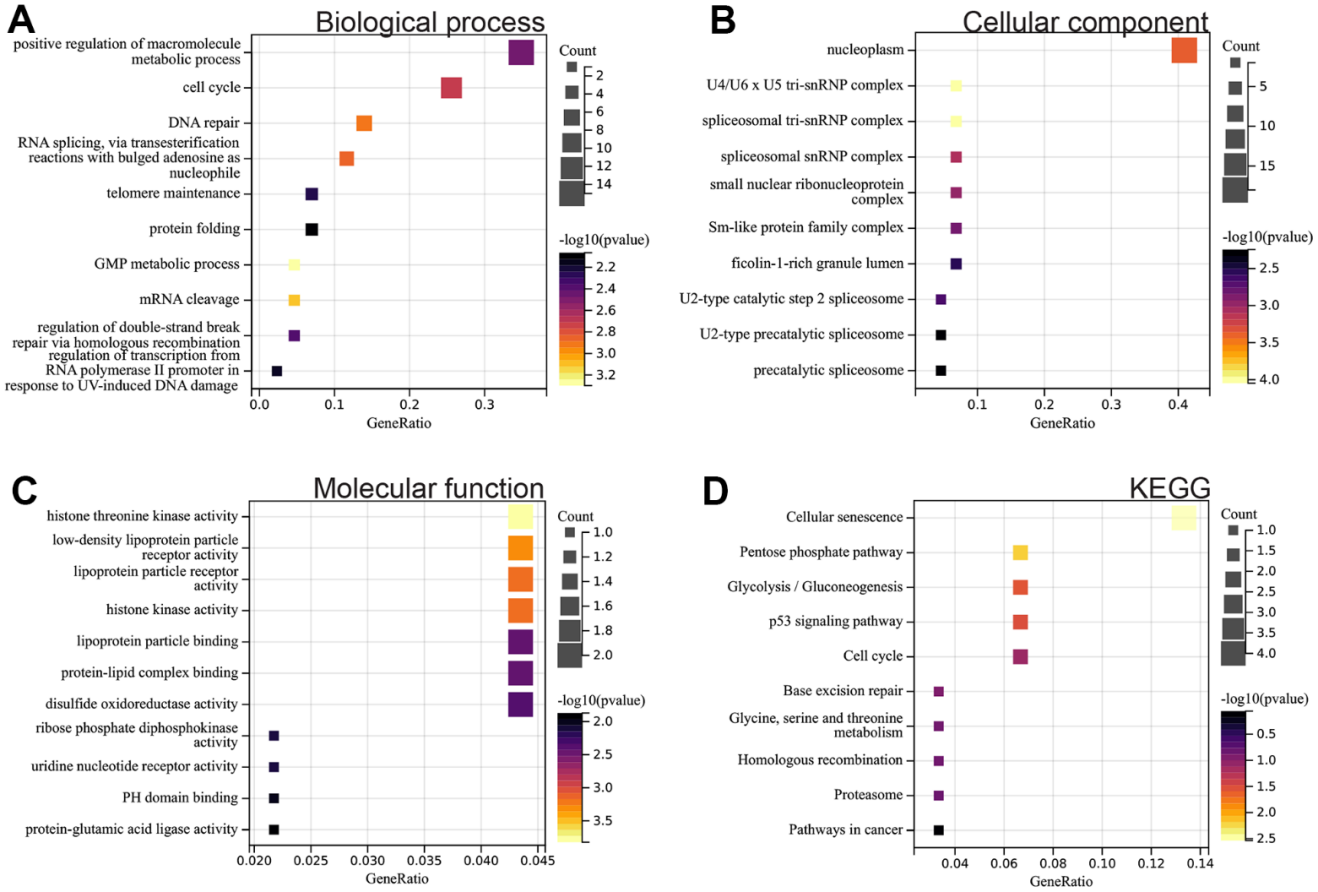
**Functional enrichment analysis.** (**A**) BP (**B**) CC (**C**) MF (**D**) KEGG analysis.

We performed GO and KEGG analyses on the identified DEGs. In the biological process (BP) category, DEGs were enriched in cell cycle, DNA repair, and protein folding (Figure 2A). In the cellular component (CC) category, they were predominantly enriched in nuclear plasma and the Sm protein family complex (Figure 2B). In the molecular function (MF) category, they showed concentration in protein-lipid complex binding and protein-glutamate ligase activity (Figure 2C). Additionally, the KEGG analysis highlighted DEGs' enrichment in pathways related to cell aging, the P53 signaling pathway, glycolysis/gluconeogenesis, cell cycle, and cancer (Figure 2D).

### GSEA

**Figure 3:**
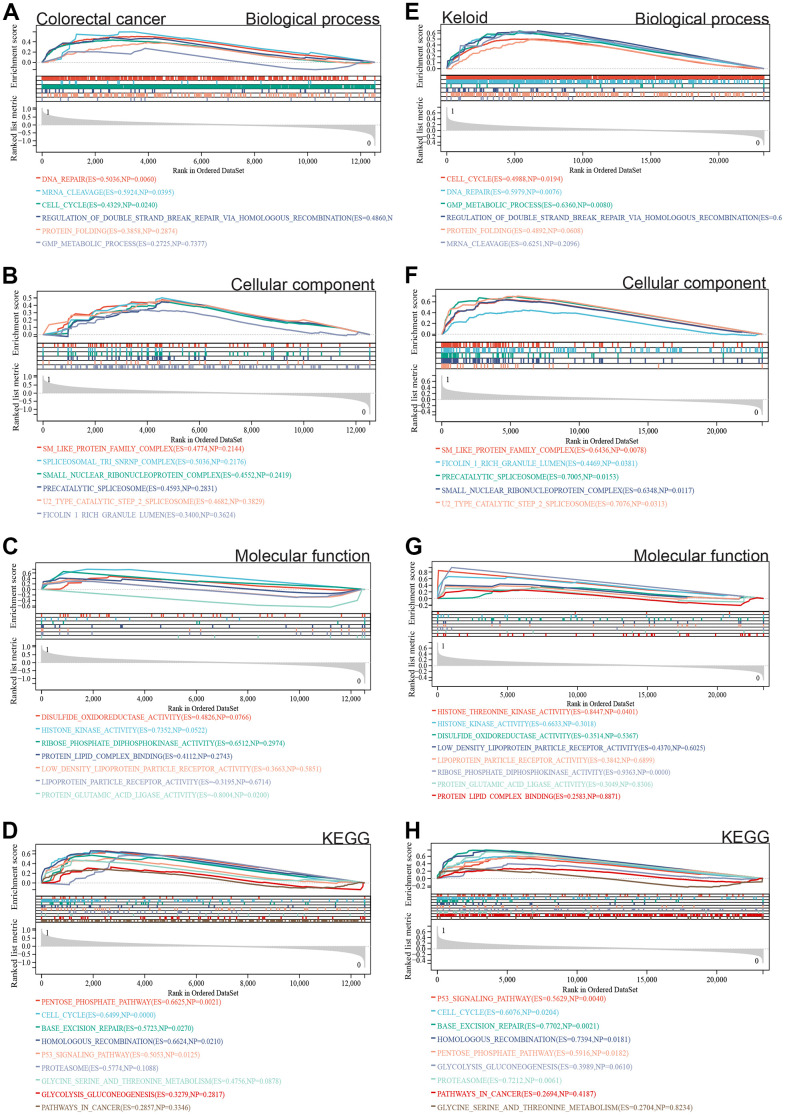
**GSEA.** (**A**–**D**) The differentially expressed genes in colorectal cancer are mainly concentrated in cell cycle, DNA repair, SM-like protein family complex, protein-lipid complex binding, P53 signaling pathway, glycolysis/gluconeogenesis, and cancer pathway. (**E**–**H**) The intersection of gene expression matrix enrichment items and differentially expressed gene GO KEGG enrichment items in keloid was similar to that in colorectal cancer.

Furthermore, we performed GSEA on the entire genome to identify potential enrichments among non-DEGs and validate the results of DEG analysis. The GSEA results for colorectal cancer (Figure 3A–3D) and scar tissue (Figure 3E–3H) showed enrichment in similar pathways to the DEG analysis, including cell cycle, DNA repair, Sm protein family complex, protein-lipid complex binding, P53 signaling pathway, glycolysis/gluconeogenesis, and cancer pathways.

### Metascape enrichment analysis

**Figure 4:**
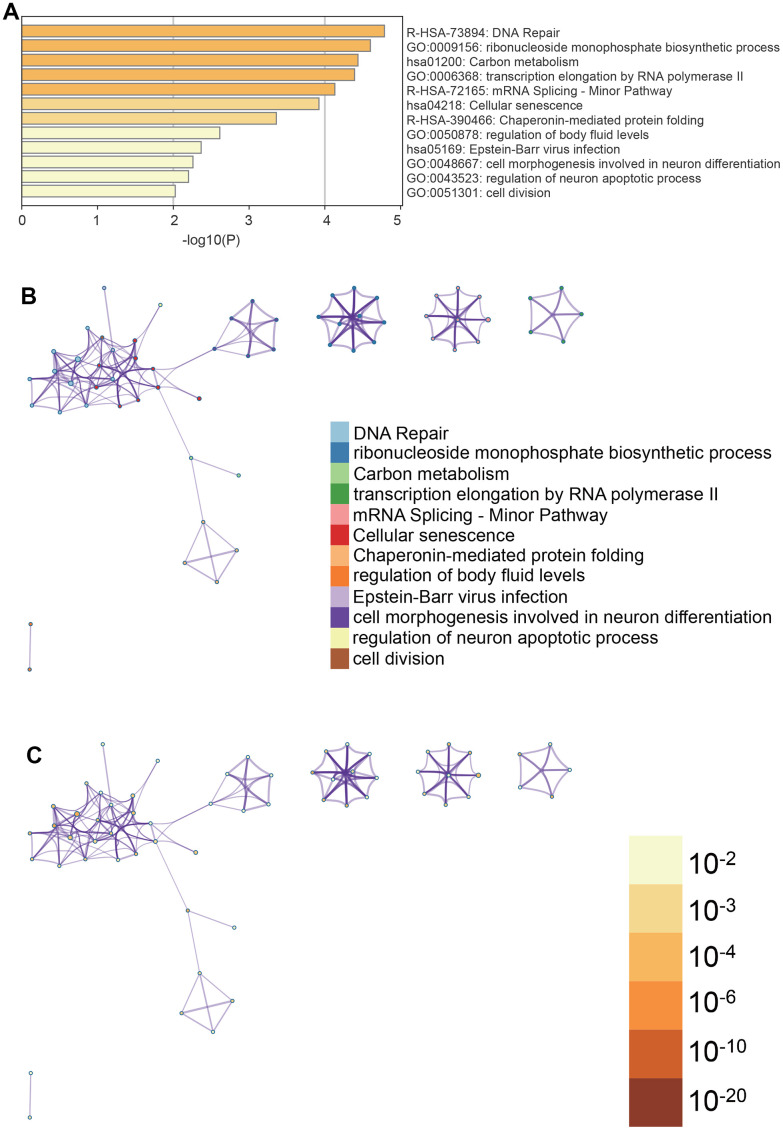
**Metascape enrichment analysis.** (**A**) Cell senescence, cell division and DNA repair can be seen in the GO enrichment project (**B**) enrichment networks colored by enrichment terms (**C**) enrichment networks colored by p values.

Metascape enrichment analysis (Figure 4A) indicated significant enrichment in terms such as cell aging, cell division, and DNA repair, among others. To better understand these enrichments, we created enrichment networks (Figure 4B, 4C) that visualize the relationships and confidence levels of each enrichment term.

### Construction and analysis of protein-protein interaction (PPI) network

**Figure 5:**
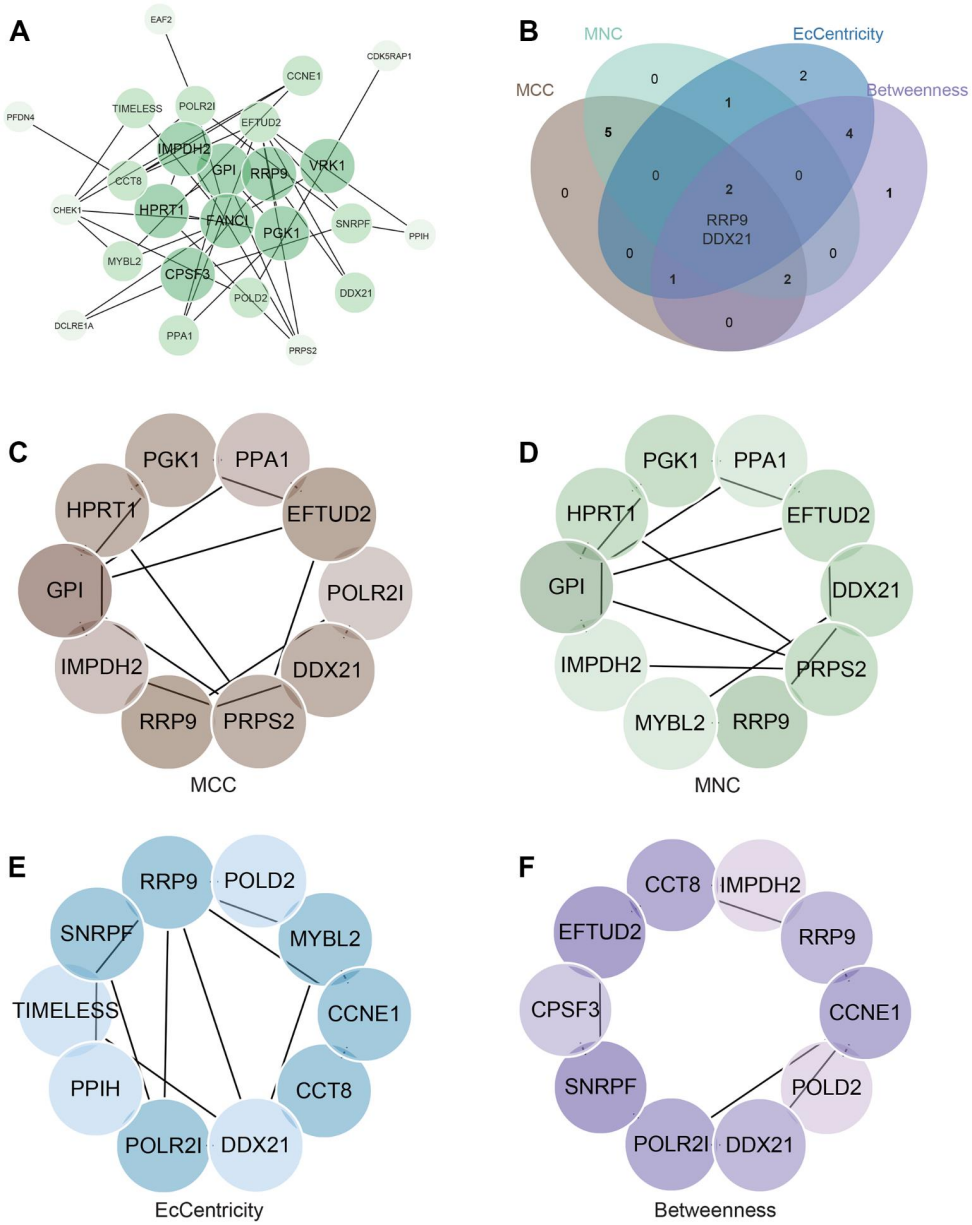
**Construction and Analysis of protein-protein interaction (PPI) Network.** (**A**) PPI network. (**B**) Four algorithms identify the central gene. (**C**–**F**) MCC, MNC, EcCentricity, Betweenness identify the central genes.

We built the PPI network of DEGs with data from the STRING database and analyzed it in Cytoscape (Figure 5A). Employing four centrality algorithms (MCC, MNC, EcCentricity, Betweenness), we identified two core genes (RRP9 and DDX21) through their intersection (Figure 5B–5F).

### Core gene expression heatmaps

**Figure 6:**
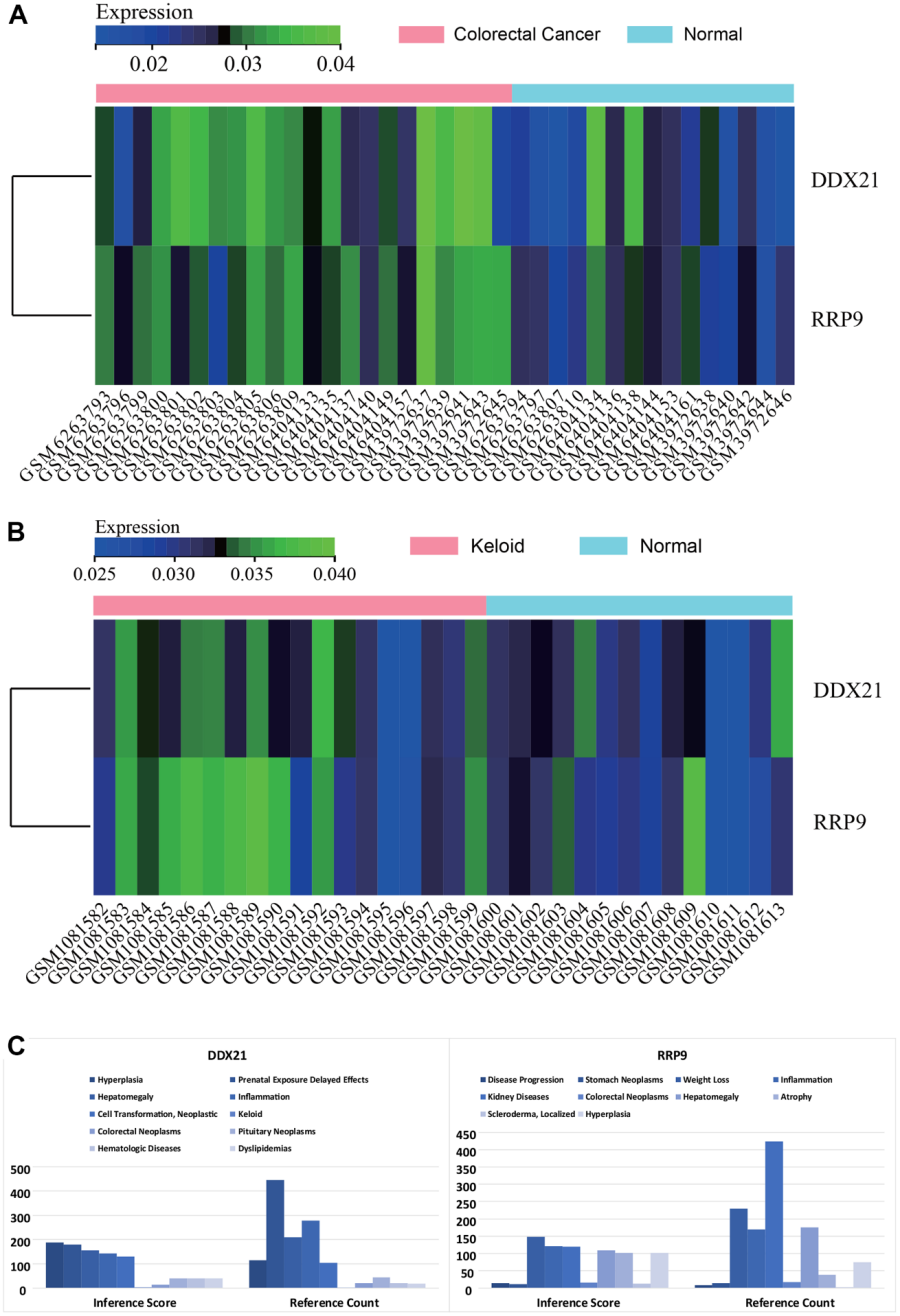
**Gene expression heat map.** (**A**) colorectal cancer. (**B**) keloids. (**C**) CTD analysis, Core genes (RRP9, DDX21) were found to be associated with hyperplasia, keloid, colorectal tumor, scleroderma, and inflammation.

Heatmaps were generated to visualize the expression levels of core genes (RRP9 and DDX21) in the batch-corrected matrix of colorectal cancer (Figure 6A) and scar tissue (Figure 6B) datasets. The core genes exhibited higher expression in colorectal cancer and scar tissue samples compared to normal samples, suggesting their potential regulatory role in colorectal cancer and scar tissue.

### CTD analysis

CTD analysis revealed associations between the core genes (RRP9 and DDX21) and diseases such as proliferation, scar tissue, colorectal tumors, scleroderma, and inflammation (Figure 6C).

### WB analysis

**Figure 7:**
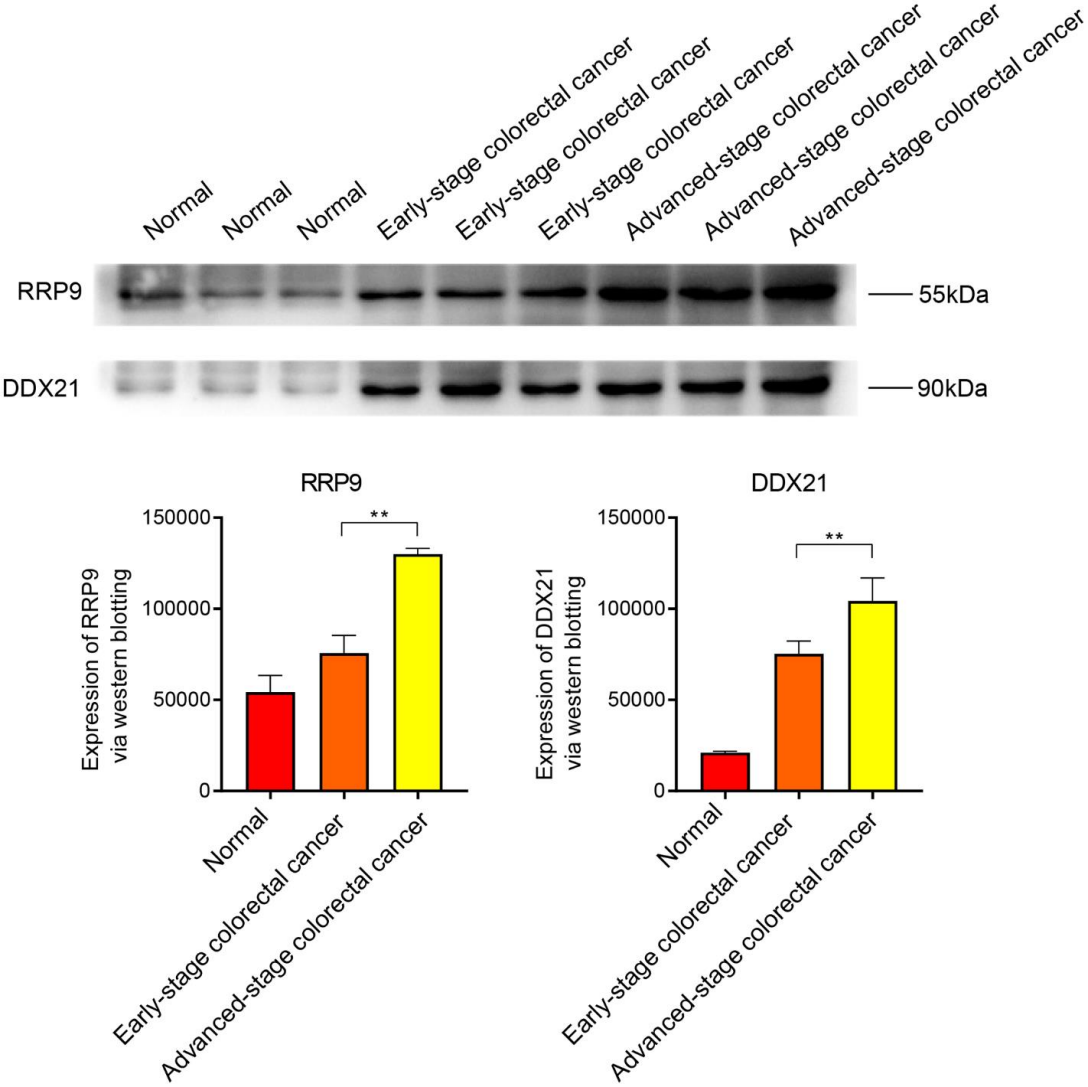
**WB experiments.** Expression levels of RRP9 and DDX21 in colorectal cancer.

**Figure 8:**
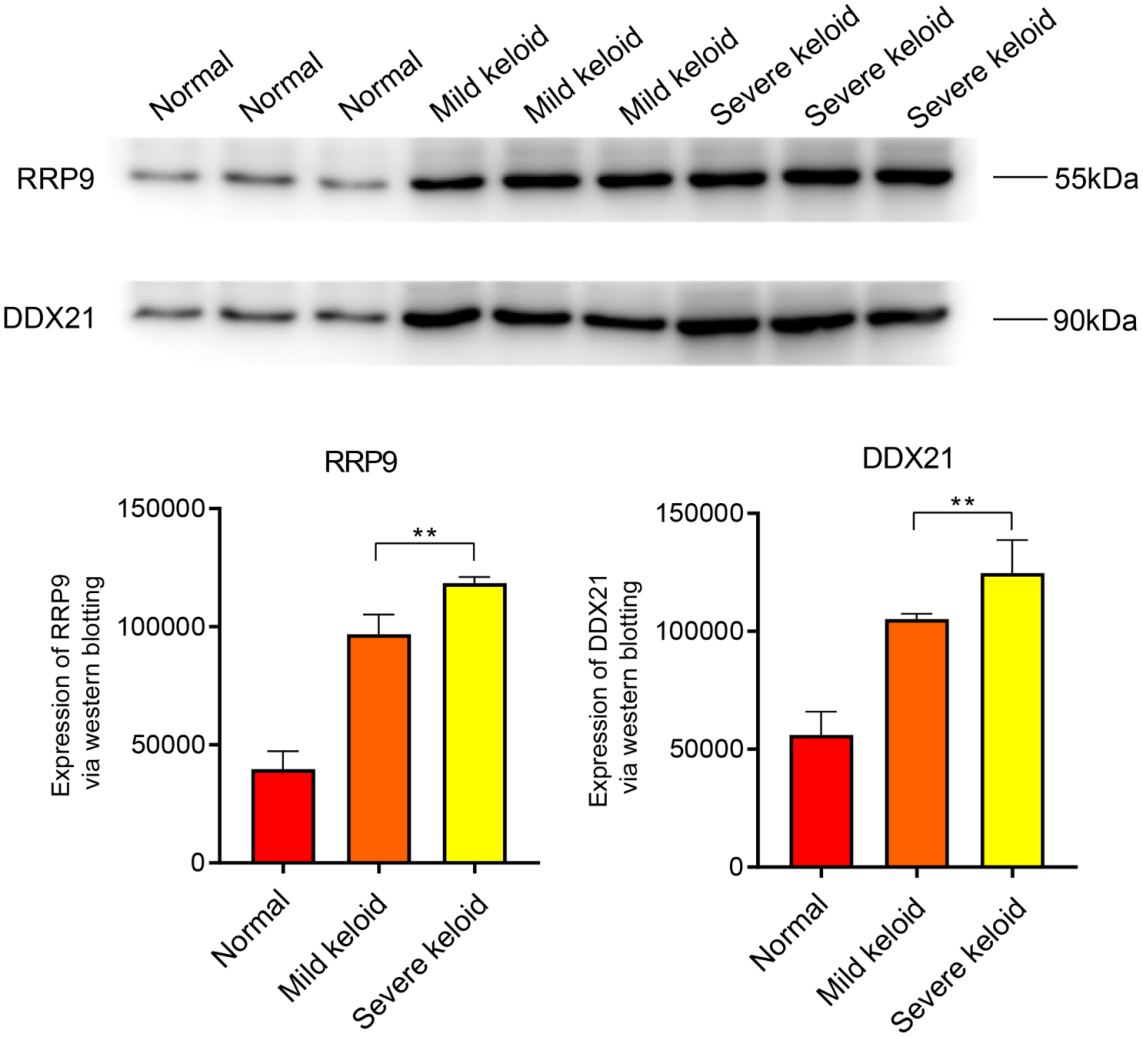
**WB experiments.** Expression levels of RRP9 and DDX21 in keloid.

The Western blot (WB) results indicate that the core genes RRP9 and DDX21 are highly expressed in both early and late-stage colorectal cancer, with a more significant expression level observed in the late-stage (Figure 7). In scar tissue, when compared to normal tissue, RRP9 and DDX21 exhibit elevated expression levels, particularly in cases of severe scar tissue (Figure 8).

### PCR analysis

**Figure 9:**
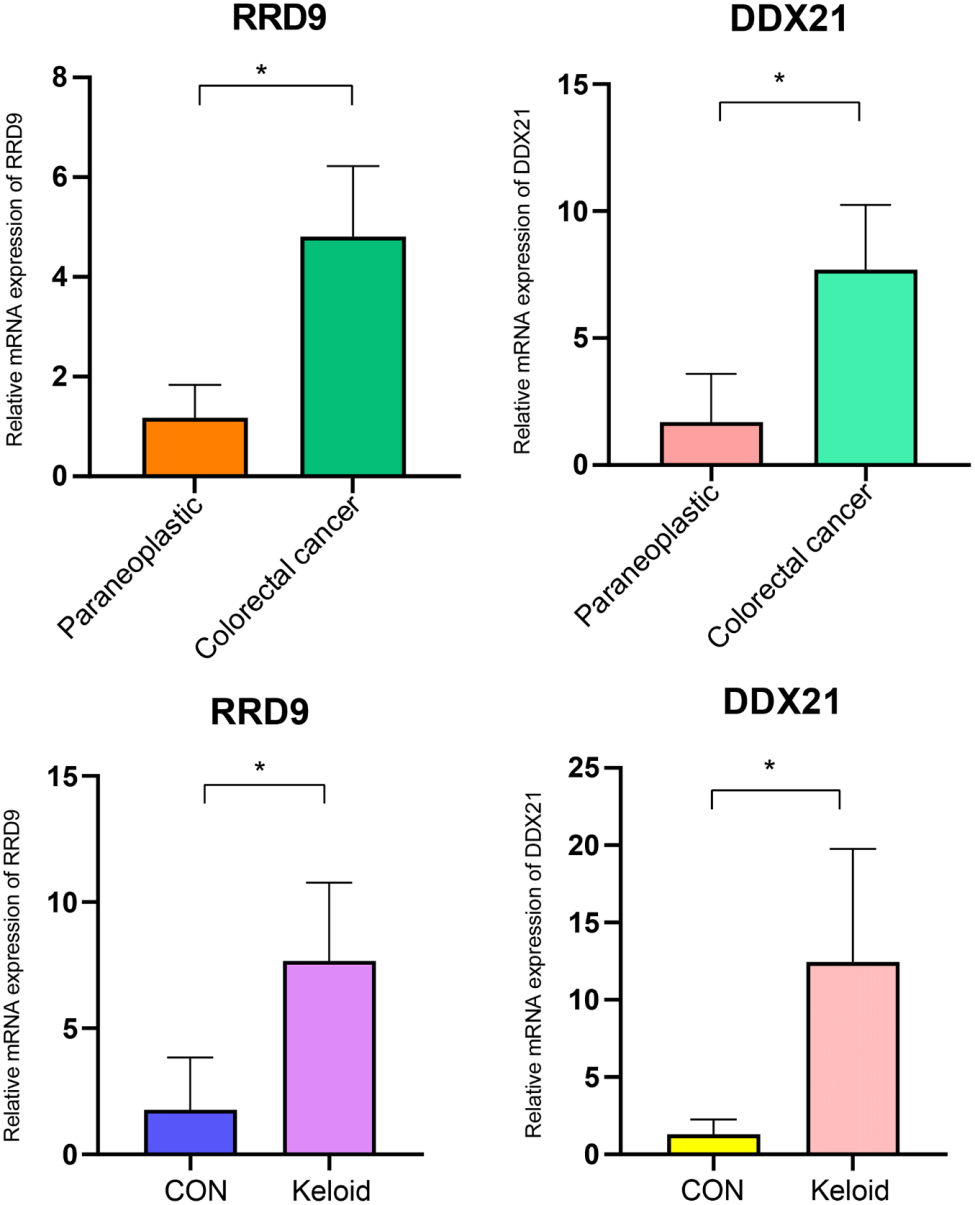
**PCR experiments.** Expression levels of RRP9 and DDX21 in colorectal cancer and keloid.

The polymerase chain reaction (PCR) results reveal that the core genes RRP9 and DDX21 are highly expressed in both colorectal cancer and scar tissue (Figure 9).

### Prediction of miRNA and functional annotation associated with hub genes

In this study, we used TargetScan to predict miRNAs associated with the hub genes. We identified hsa-miR-374c-5p and hsa-miR-655-3p as miRNAs associated with RRP9, and hsa-miR-218-5p as a miRNA associated with DDX21 (Table 1).

**Table 1:** A summary of miRNAs that regulate hub genes.

	**Gene**	**MIRNA**	
1	RRP9	hsa-miR-374c-5p	hsa-miR-655-3p
2	DDX21	hsa-miR-218-5p	

## DISCUSSION

Colorectal cancer is a malignant tumor originating from the tissues of the colon or rectum [18]. Colorectal cancer ranks among the top causes of cancer-related fatalities globally. In its early stages, colorectal cancer often lacks noticeable symptoms, resulting in late detection that poses greater treatment challenges. The danger of this disease lies in its ability to grow rapidly, invade surrounding tissues, and potentially spread to other organs, posing a significant threat to the patient's life [19, 20]. Keloids, also known as scar hypertrophy, are abnormal skin lesions typically resulting from skin damage or trauma during the healing process [21]. Although not typically life-threatening, keloids can significantly diminish the patient's quality of life. They can lead to itching, pain, discomfort, and alterations in physical appearance, affecting the patient's confidence and psychological well-being. Gaining a more profound insight into the molecular mechanisms of colorectal cancer can reveal key factors and signaling pathways that propel the cancer's progression. This can aid in the discovery of new therapeutic targets and the design of more effective targeted drugs [22]. The results of this study indicate that RRP9 and DDX21 are overexpressed in both colorectal cancer and keloids, and their high expression is associated with worsened prognosis. These two genes exert their effects on colorectal cancer and keloids' prognosis by influencing various cellular and molecular processes, including cell proliferation, extracellular matrix remodeling, and inflammatory responses.

Ribosomal biogenesis requires coordination of small nucleolar ribonucleoprotein (snoRNP) and ribosomal proteins [23]. SnoRNAs primarily engage in post-transcriptional RNA modification and cellular RNA maturation [24]. Since cell growth requires the production of new ribosomes, cancer cells use them to support accelerated growth [25]. Indeed, research has demonstrated the potential carcinogenic role of snoRNA in various cancers. RRP9 serves as a central component of the U3-snoRNP complex and plays a role in pre-rRNA processing [26]. Diacylation modification tests established RRP9's association with Nedd8. Smurf1 catalyzes the Neddylation of RRP9, a process reversed by NEDP1 adenosinase. Notably, Lys221 was verified as the principal Neddylated Nadierization site in RRP9. The absence of RRP9 Neddylation modification impedes pre-rRNA processing, resulting in reduced ribosome biogenesis. Furthermore, heightened RRP9 and Smurf1 expression correlates with the progression of human colorectal cancer [15, 27, 28]. Zhang et al. found that the expression of U3snoRNA-related protein RRP9/U3-55K was significantly increased in the human pancreatic tissue. RRP9 is identified as a new target and may be beneficial to the treatment of PC. RRP9 plays an indispensable role in promoting chemical resistance to gemcitabine. During the development of keloids, various characteristics such as inflammation are involved [29]. RRP9 may also play a significant role in extranuclear cellular processes, which relates to the inflammatory response. RRP9 might influence the inflammatory response by interacting with proteins or pathways related to the immune system, which includes regulating the activation or inhibition of white blood cells. Additionally, RRP9 regulates the expression levels of genes associated with inflammation by affecting gene expression, achieved through its role in transcription or post-transcriptional regulation. Higher expression levels of RRP9 are associated with a more unfavorable impact on keloids. Therefore, targeting RRP9 may provide a potential therapeutic strategy. The above literature review is consistent with our results. The higher the expression of RRP9 in colorectal cancer, the higher the RRP9, the worse the prognosis.

The DExD-box helicase 21 (DDX21) protein is an RNA helicase involved in several cellular processes, such as altering RNA secondary structures and engaging in nuclear and mitochondrial splicing [30]. DDX21 plays various rolesin ribosomes such as transcription and RNA processing [31]. DDX21 effectively unlocks R ring and prevents R-loop-mediated RNA polymerase arrest, whereas DDX21 deletion results in cellular R ring accumulation and DNA damage. Over the past decade, DDX21 has Garnered much attention for its role in tumors. Related studies have found that down-regulation of the long non-coding RNA (lncRNA) HCP5 can restrict the proliferation, migration, and invasion of gastric cancer cells by regulating expression level of DDX21 [32, 33]. Tanaka et al. performed unbiased proteomic analysis of a colorectal cancer cohort using mass spectrometry and showed that DDX21 expression was upregulated in cancer tissues. Additionally, the expression of DDX21 protein was examined in the verification group of 710 patients, including 619 with early colorectal cancer and 91 with advanced colorectal cancer. DDX21 was mainly expressed in the tumor nucleus and highly expressed in some mitotic cells. The level of DDX21 expression was associated with non-mucous histology in early cancer. Survival analysis showed that high DDX21 expression was associated with the survival of the patients with early colorectal cancer [34]. The research by Yanzhu et al. showed that the expression level of DDX21 in colorectal cancer tissues is higher than that in normal tissues [35]. In the development of keloids, the regulation of RNA synthesis and stability may be involved, and DDX21 is suggested to play a role in these processes. Higher expression levels of DDX21 may lead to decreased RNA stability, which can subsequently impact the prognosis. The literature reviewed above is consistent with the findings of our study, where increased expression of DDX21 is associated with a poorer prognosis in both colorectal cancer and keloids. Hence, we speculate that DDX21 plays a crucial role in these two diseases. The above relevant literature review is consistent with the results of this study. The higher expression of DDX21 in colorectal cancer and keloids, the higher the DDX21, the worse the prognosis. We speculate that DDX21 may have an important effect on colorectal cancer and keloids.

## CONCLUSIONS

Signal proteins and pathways are typically vital targets in cancer therapy. RRP9 and DDX21 exhibit high expression and significant roles in patients suffering from colorectal cancer and keloids. RRP9 and DDX21 may represent molecular targets for both diseases. Further investigation into their functions and their connection to signaling pathways could reveal their potential significance in the diagnosis, prognosis, and treatment of colorectal cancer and keloids.

## MATERIALS AND METHODS

### Colorectal cancer and scar tissue dataset

In this study, we downloaded colorectal cancer datasets GSE134834, GSE206800, GSE209892, and scar tissue dataset GSE44270 from the GEO database (http://www.ncbi.nlm.nih.gov/geo/). GSE134834 has 5 CRC and 5 normal tissue samples, GSE206800 includes 11 CRC and 4 normal tissue samples, GSE209892 comprises 6 CRC and 6 normal tissue samples, and GSE44270 has 18 scar tissue and 14 normal tissue samples. These datasets were used to identify DEGs between colorectal cancer and scar tissue.

### Batch correction

For data integration and batch correction across multiple datasets, we initially merged the colorectal cancer datasets GSE134834, GSE206800, and GSE209892 using the R package *in Silico* Merging and obtained a merged matrix. Further, we employed the R package limma (version 3.42.2) and its remove BatchEffect function to remove batch effects, resulting in a batch-effect-corrected matrix that was used for subsequent analyses.

### DEG selection

We performed log2 transformation on the batch-corrected merged matrices of the colorectal cancer datasets GSE134834, GSE206800, and GSE209892, as well as the gene expression matrix of the scar tissue dataset GSE44270. We then conducted multivariate linear regression using the lmFit function. After adjusting the standard errors towards a common value using empirical Bayesian methods, we calculated moderated t-statistics, moderated f-statistics, and log-fold changes for differential expression of each gene. This process enabled us to identify significantly differentially expressed genes (DEGs) and create volcano plots. Subsequently, we obtained the intersection of DEGs between the colorectal cancer datasets and the scar tissue dataset.

### Functional enrichment analysis

We employed Gene Ontology (GO) and Kyoto Encyclopedia of Genes and Genomes (KEGG) analyses for gene function and pathway assessment. Gene annotation was obtained from the latest KEGG Pathway via the KEGG REST API (https://www.kegg.jp/kegg/rest/keggapi.html) as the background for gene mapping. Enrichment analysis was performed using the R package clusterProfiler (version 3.14.3) with a significance threshold of P < 0.05 and a false discovery rate (FDR) of < 0.25, while setting the minimum gene set size to 5 and the maximum gene set size to 5000.

Additionally, we conducted functional enrichment analysis via the Metascape database (http://metascape.org/gp/index.html) for annotating and visualizing DEGs.

### Gene set enrichment analysis (GSEA)

In our Gene Set Enrichment Analysis (GSEA), we categorized samples as either “disease” (colorectal cancer) or “normal” (scar tissue). We used GSEA software (version 3.0) from the Broad Institute and obtained the c2.cp.kegg.v7.4.symbols.gmt subset from the Molecular Signatures Database. GSEA was employed to investigate relevant pathways and molecular mechanisms based on gene expression profiles and sample classification. We set specific parameters, including a minimum gene set size of 5, a maximum gene set size of 5000, performed 1000 permutations, and considered results statistically significant with a P value < 0.05 and FDR < 0.25. Additionally, we conducted comprehensive GO and KEGG analyses across the entire genome using GSEA.

### Construction and analysis of protein-protein interaction (PPI) network

We constructed the Protein-Protein Interaction (PPI) network of DEGs by utilizing the STRING database (http://stringdb.org/), which consolidates protein interaction data from diverse sources. To analyze and visualize the PPI network, we employed Cytoscape software. For identifying the most significant modules within the network, we applied the MCODE algorithm. Additionally, we calculated centrality scores using four algorithms (MCC, MNC, EcCentricity, Betweenness) to identify the top ten genes with the highest centrality scores. The genes at the intersection of these rankings were extracted as the list of core genes and visually presented.

### Gene expression heatmaps

We generated heatmaps of core gene expression using the R package “heatmap”. These heatmaps showcased core gene expression in both the batch-corrected matrix of colorectal cancer and the gene expression matrix of scar tissue. This visualization highlighted expression distinctions among colorectal cancer, scar tissue, and normal samples.

### CTD analysis

The Comparative Toxicogenomics Database (CTD) consolidates information regarding interactions among chemicals, genes, phenotypes, and diseases, offering valuable insights into gene-disease relationships. We utilized the CTD website to input the list of hub genes and identify diseases associated with the core genes. Radar plots were created in Excel to visually depict the expression variations of each gene.

### miRNA

We employed TargetScan (https://www.targetscan.org), an online database, to predict miRNAs responsible for regulating the core DEGs identified in our study.

### Reverse transcription polymerase chain reaction (RT-PCR)

We analyzed 40 tissue samples, including 20 adjacent tissue samples and 20 colorectal cancer tissue samples, obtained from the Department of General Surgery, The Fourth Affiliated Hospital, Hebei Medical University. The study was approved by the Ethics Committee of the Fourth Affiliated Hospital of Hebei Medical University (No. 2021KY039), and the consent was signed by the patients. Participants were characterized by gender (10 males and 10 females), age (10 individuals older than 60), smoking habits (9 smokers and 11 non-smokers), alcohol consumption (evenly distributed), and family history (present/absent). In addition, normal and keloid tissues were also examined. We conducted Reverse Transcription Polymerase Chain Reaction (RT-PCR) on these specimens with the following steps:

(1) Reverse Transcription: RNA templates were converted into complementary DNA (cDNA) strands using reverse transcriptase. (2) Polymerase Chain Reaction (PCR): We amplified cDNA using PCR, involving cycles of heating and cooling to generate abundant target DNA. (3) Amplification Cycles: Each cycle included denaturation (heating to separate DNA strands), annealing (primer binding to target sequences), and extension (DNA polymerase synthesizing new DNA strands). (4) Termination of Reaction: A final cooling step ensured complete DNA amplification and storage at an appropriate temperature. (5) Result Analysis: Gel electrophoresis determined the presence and quantity of specific DNA sequences in the amplified DNA.

### Western blotting (WB)

In our experiment, we used colorectal cancer cell lines (1101HUM-PUMC000109) and normal colon cells (5301HUM-KCB18026YJ) as samples. In addition, normal and keloid tissues were also examined. Here are the specific experimental steps.

(1) Protein Quantification: Extract total proteins and measure the protein concentration in the samples using the Bradford assay. (2) SDS-PAGE Gel Preparation: Prepare a polyacrylamide gel, cast the gel, and allow it to polymerize. (3) Sample Denaturation and Loading: Mix the protein samples with sample buffer containing SDS and a reducing agent, heat the samples to denature the proteins, and then load them into the gel wells. (4) Electrophoresis: Perform electrophoresis to separate proteins. (5) Membrane Transfer: After electrophoresis, use a wet or semi-dry transfer apparatus to transfer the separated proteins from the gel onto a nitrocellulose or PVDF membrane. This step preserves the protein pattern from the gel on the membrane. (6) Blocking: Incubate the membrane with a blocking solution (5% skim milk). (7) Primary Antibody Incubation: Incubate the membrane with a primary antibody specific to the target protein overnight at 4° C or at room temperature. (8) Washing: Wash the membrane to remove unbound primary antibody, typically with buffer solution through multiple washes. (9) Secondary Antibody Incubation: Incubate the membrane with an enzyme-linked secondary antibody (HRP-conjugated secondary antibody) specific to the primary antibody. (10) Washing: Wash the membrane again to remove unbound secondary antibody. (11) Detection: Add a substrate suitable for the enzyme on the secondary antibody (HRP). The enzyme catalyzes a chemical reaction that produces chemiluminescence (12) Image Capture: Capture images of the chemiluminescence using a specialized imaging system (fluorescence imager).

### Data availability

The datasets generated during and/or analyzed during the current study are available from the corresponding author on reasonable request.
